# Possible association of elevated CSF IL‐6 levels with anxiety and frustration in psychiatric disorders

**DOI:** 10.1111/pcn.13743

**Published:** 2024-09-24

**Authors:** Takako Enokida, Kotaro Hattori, Kaori Okabe, Takamasa Noda, Miho Ota, Noriko Sato, Shintaro Ogawa, Megumi Tatsumi, Mikio Hoshino, Hiroshi Kunugi, Kazuyuki Nakagome

**Affiliations:** ^1^ Department of Bioresources, Medical Genome Center National Center of Neurology and Psychiatry Tokyo Japan; ^2^ Department of NCNP Brain Physiology and Pathology, Cognitive and Behavioral Medicine, Graduate School of Medical and Dental Sciences Tokyo Medical and Dental University Tokyo Japan; ^3^ Department of Psychiatry National Center Hospital, National Center of Neurology and Psychiatry Tokyo Japan; ^4^ Department of Radiology, National Center Hospital National Center of Neurology and Psychiatry Tokyo Japan; ^5^ Department of Neuropsychiatry University of Tsukuba Ibaraki Japan; ^6^ Department of Mental Disorder Research, National Institute of Neuroscience National Center of Neurology and Psychiatry Tokyo Japan; ^7^ Department of Biochemistry and Cellular Biology, National Institute of Neuroscience National Center of Neurology and Psychiatry Tokyo Japan; ^8^ Department of Psychiatry Teikyo University School of Medicine Tokyo Japan

**Keywords:** cerebrospinal fluid, cerebrovascular circulation, interleukin‐6, Neuroinflammatory diseases

## Abstract

**Aim:**

Neuroinflammation is an important causal factor for a variety of psychiatric disorders. We previously reported increased cerebrospinal fluid interleukin‐6 levels in patients with schizophrenia and major depressive disorder. The present study aimed to examine the possible association of interleukin‐6 levels with anxiety and frustration, negative valence symptoms shared in various psychiatric disorders.

**Methods:**

We included 129 patients with psychiatric disorders and 70 controls. CSF and plasma interleukin‐6 levels were measured by immunoassay kits, and psychological symptoms were assessed with the State–Trait Anxiety Inventory, and the Basic Psychological Need Satisfaction and Frustration Scale. To examine regional cerebral blood flow, patients underwent arterial spin labeling analysis using magnetic resonance imaging.

**Results:**

Cerebrospinal fluid interleukin‐6 levels were significantly correlated with State–Trait Anxiety Inventory‐trait anxiety (*r* = 0.25, *P* = 0.046) and Basic Psychological Need Satisfaction and Frustration Scale‐autonomy frustration scores (*r* = 0.29, *P* = 0.018). Patients with abnormally high cerebrospinal fluid interleukin‐6 levels (defined >97.5 percentile of the controls) had higher scores for trait anxiety (*P* = 0.035) and autonomy frustration (*P* = 0.026), and significantly increased regional cerebral blood flow in the left superior temporal gyrus, bilateral nucleus accumbens, and cerebellum than the remaining patients.

**Conclusion:**

Patients with elevated cerebrospinal fluid interleukin‐6 constitute a subpopulation of psychiatric disorders associated with anxiety and autonomy frustration, which may be related to altered functions in specific brain areas.

Traditionally, studies of psychiatric disorders have assumed a specific brain pathology for each disorder. However, the heterogeneity and comorbidity of the disorders make it difficult to identify a specific pathology, and it is now believed that treatment should target underlying symptoms and dysfunctions rather than the disease itself. Transdiagnostic analyses that focus on symptoms rather than disease may identify common biomarkers and therapeutic targets.

Neuroinflammation has been implicated in a variety of psychiatric disorders. In line, interleukin‐6 (IL‐6) is a major proinflammatory cytokine and elevated IL‐6 levels in CSF have been reported in mood disorders,^1^, ^2^ schizophrenia,^1^, ^3^ and post‐traumatic stress disorder.^4^ We also reported increased cerebrospinal (CSF) levels of IL‐6 in patients with schizophrenia and major depressive disorder (MDD).^5^ Engler *et al*. reported that low‐dose intravenous lipopolysaccharide (LPS) administration to healthy participants selectively increased CSF IL‐6 levels after peripheral inflammation, and a strong association between CSF IL‐6 levels and mood symptoms.^6^ Thus, an increase in CSF IL‐6 may play an important causal role across different psychiatric disorders.

The Research Domain Criteria project (RDoC), initiated at the National Institute of Mental Health (NIMH) in 2009, proposed a new framework for psychiatric research based on genetics, neurobiology, and behavioral observations.^7^, ^8^ The RDoC is expected to advance the study of psychopathology beyond the limitations of symptom‐based diagnoses. In fact, transdiagnostic research approaches based on RDoC have been growing.^9^, ^10^

Negative valence systems (NVS) are a functional domain of RDoC, and are primarily responsible for reactions to aversive situations or context.^11^ NVS consists of five components: acute threat (fear), potential harm (anxiety), sustained threat, loss, and frustrative non‐reward. Among these components, we focused on potential harm (anxiety) and frustrative non‐reward because these components are commonly observed in various psychiatric disorders and well‐validated self‐report measures are available. In the NVS context, ‘anxiety’ is the activation of a brain system characterized by enhanced risk assessment in low imminent threats; ‘frustrative non‐reward’ is a reaction elicited by the inability to obtain positive rewards after repeated or sustained efforts, and has been examined as a measure of aggression.^12^, ^13^ Dysregulation of reward systems has also been implicated in mood disorders.^14^

The present study aimed to examine whether CSF IL‐6 levels are associated with anxiety and frustrative non‐reward. In addition, we examined the possible association of CSF IL‐6 levels with peripheral inflammation markers, blood–brain barrier integrity, and regional cerebral blood flow (rCBF).

## Methods

### Participants and clinical assessments

Participants were recruited at the National Center of Neurology and Psychiatry (NCNP) Hospital, Tokyo, Japan, through website announcements. All the participants were East Asians and most of them were Japanese. Participants with a history of CNS disease or severe head injury were excluded. The control participants underwent the Mini‐International Neuropsychiatric Interview (M.I.N.I.) to screen for psychiatric disorders.^15^ Participants with psychiatric disorders were trans‐diagnostically recruited, and underwent symptom assessment interviews, including the Structured Clinical Interview for DSM–IV‐TR Axis I Disorder (SCID‐I),^15^, ^16^ self‐report assessment using State–Trait Anxiety Inventory (STAI),^17^ and Basic Psychological Need Satisfaction and Frustration Scale (BPNSFS),^18^, ^19^ and magnetic resonance imaging (MRI) of the brain. The medication status of patients at the time of lumbar puncture was recorded. Daily doses of antipsychotics, antidepressants, and benzodiazepines were converted to equivalent doses of chlorpromazine, imipramine, and diazepam, respectively, according to the published guideline.^20^ To evaluate the disease severity, patients with psychiatric disorders underwent the Japanese Adult Reading Test (JART),^21^ Positive and Negative Syndrome Scale (PANSS),^22^ Montgomery–Åsberg Depression Rating Scale (MADRS),^23^ and clinical evaluation of manic disorders using the Japanese version of the Young Mania Rating Scale (YMRS).^24^

This study was conducted in accordance with the Declaration of Helsinki^25^ and was approved by the ethics committee of NCNP, Japan (A2012‐091 for biobanking, A2016‐005 for NVS evaluation, and A2019‐092 for CSF collection and biomarker analyses). Written informed consent was obtained from all participants.

### CSF and blood sample collection

Both CSF and plasma samples were collected from the NCNP biobank as described previously.^26^ None of the samples in the present study overlapped with those used in our previous study^5^ on increased CSF IL‐6 levels in patients with schizophrenia and MDD.^5^

### Laboratory examination of CSF

All CSF samples were screened using the general laboratory test for cell count, glucose, and total protein (TP), and samples within the normal range were used for analysis. A sample with an abnormally high cell count (>500 /μL) was excluded from the analysis.

### IL‐6 measurement

CSF IL‐6 concentrations were measured at Eurofins GeneticLab Co., Ltd. (Sapporo, Japan) using Bio‐Plex Pro™ Human Inflammation Panel 1, 3‐plex (Bio‐Rad Laboratories, Inc., Hercules, CA), and Luminex® 100/200™ System (Luminex Corp. Austin, TX). The CSF samples were diluted 1:2 and measured individually. Between‐plate normalization was performed using bridging samples (n = 16) from each plate. Plasma IL‐6 and C‐reactive protein (CRP) concentrations were measured at Acel, Inc. (Tokyo, Japan), using a Human Premixed Multi‐Analyte Kit (R&D Systems, Inc. MN) according to the manufacturer's instructions on a Bio‐Plex 200 system (Bio‐Rad Laboratories, Inc.) with low‐photomultiplier tube settings. Analyte concentrations were calculated using the Bio‐Plex Manager software (version 6.2.0.175, Bio‐Rad Laboratories, Inc.). The coefficient of variation of overlapping samples of CSF IL‐6, plasma IL‐6 and plasma CRP were 5.6%, 7.1%, and 3.5%, respectively, indicating the accuracy of these measurements.

### MRI data acquisition and processing

MRI was performed using a 3 Tesla MR system (Philips Medical Systems, Best, Netherlands). The imaging parameters for all of the 3D‐pseudo‐continuous arterial spin labeling (pCASL) experiments were identical: single‐shot gradient‐echo echo planar imaging (EPI) in combination with parallel imaging (SENSE factor 2.0), FOV = 240 × 240, matrix = 80 × 80, voxel size = 3.0 × 3.0 mm, 52 slices acquired in ascending order, slice thickness = 3 mm with no interslice gap, labeling duration = 1650 ms, post spin labeling delay = 1800 ms, TR = 5716 ms, TE = 20.5 ms, no time interval between consecutive slice acquisitions, radiofrequency (RF) duration = 0.7 ms, pause between RF pulses = 0.7 ms, labeling pulse flip angle = 25°, bandwidth = 2.2 kHz/pixel, echo train length = 100. Four pairs of control and labeled images were acquired and averaged. The scan duration was 5:27.

To measure the magnetization of the arterial blood for segmentation purposes, an EPI M0 image was acquired separately, with the same geometry and imaging parameters as the pCASL without labeling. Details of the post‐processing of arterial spin labeling (ASL) data have been previously described.^27^ The cerebral blood flow (CBF) maps were normalized using the reference template ‘SPECT.nii,’ which is the standard image for SPM8. Each map was spatially smoothed with a 6‐mm full‐width at half‐maximum Gaussian kernel to decrease spatial noise and compensate for the inexactitude of normalization.

### Statistical analysis

The Kolmogorov–Smirnov test was used to assess the normal distribution. Age, body mass index (BMI), and CSF TP levels were compared between the patients and controls using an unpaired t‐test. Sex differences were compared using the Chi‐square test. The Mann–Whitney U test was used for non‐parametric comparisons between two groups. The Kruskal–Wallis test was used to compare the median values among the diagnostic groups. Spearman's test was used to analyze the correlation between molecular levels, between molecular levels and psychotropic drug equivalents, and between psychological symptoms and disease severity scales. Partial correlation analysis with age, sex, BMI, and JART scores as control variables was used to analyze the correlations between IL‐6 levels and clinical psychological scales. The JART was used as a control variable for the effect of differences in intellectual ability on psychological scales. The cut‐off value for IL‐6 levels was set at 97.5% of the control group.^28^ Psychological scales were compared between the high and non‐high CSF IL‐6 groups by the Mann–Whitney U test. Statistical significance was set at *P* < 0.05. All analyses were conducted using SPSS (version 29, IBM Inc., Armonk, NY).

MRI statistical analyses were conducted using the SPM8 software. Differences in CBF between the two groups were assessed using a two‐sample t‐test, with age and sex as nuisance variables. Spearman's test was used to analyze the correlations between CBF and the psychological scales. Statistical significance was set at a seed level of *P* < 0.001 (uncorrected) and a cluster level of *P* < 0.05 (uncorrected), according to the statistical methods of our previous study.^29^

## Results

### Participant characteristics

We included 129 patients with psychiatric disorders and 70 healthy controls. The demographic and clinical data of the participants are presented in Table 1 and Supplementary Table S1. Patients with psychiatric disorders were classified according to DSM‐IV,^16^ including 49 with MDD, 31 with bipolar disorder, 30 with schizophrenia, and 19 with other diagnoses (two schizoaffective disorder; two delusional disorder; two psychotic disorder not otherwise specified; one depressive disorder not otherwise specified; one dysthymic disorder; one panic disorder; one social phobia; one pain disorder; one dissociative disorder; one attention‐deficit and disruptive behavior disorder; two alcohol dependence/abuse; and four adjustment disorder). There was no significant difference in age or sex between the patients and controls.

**Table 1:** Comparisons in demographic data and inflammatory marker levels between psychiatric patients and controls

	Psychiatric patients	Controls	*P*‐value
Number	129	69	–
Age	39.5 ± 12.7	42.8 ± 13.1	0.099^†^
Sex, male (%)	64 (49.6%)	35 (50.7%)	0.81^‡^
BMI	23.1 ± 3.6	22.4 ± 3.3	0.17^†^
CSF IL‐6 (pg/mL)	1.3 (0.9–2.0)	1.1 (0.8–1.8)	0.055^§^
PL IL‐6 (pg/mL)	8.5 (6.8–11.2)	8.5 (7.1–10.1)	0.57^§^
PL CRP (pg/mL)	0.54 (0.5–0.57)	0.53 (0.49–0.56)	0.54^§^
CSF TP (pg/mL)	39.0 (34–49)	36.0 (30–42)	0.002^†^
JART	108 (101–113)	–	–

Abbreviations: BMI, body mass index; CRP, C‐reactive protein; CSF, cerebrospinal fluid; JART, Japanese adult reading test; PL, plasma; TP, total protein.

^†^ *T* Test;

^‡^ χ^2^ test;

^§^ Mann–Whitney U test.

### Inflammatory biomarker levels in the CSF and plasma

**Fig. 1:**
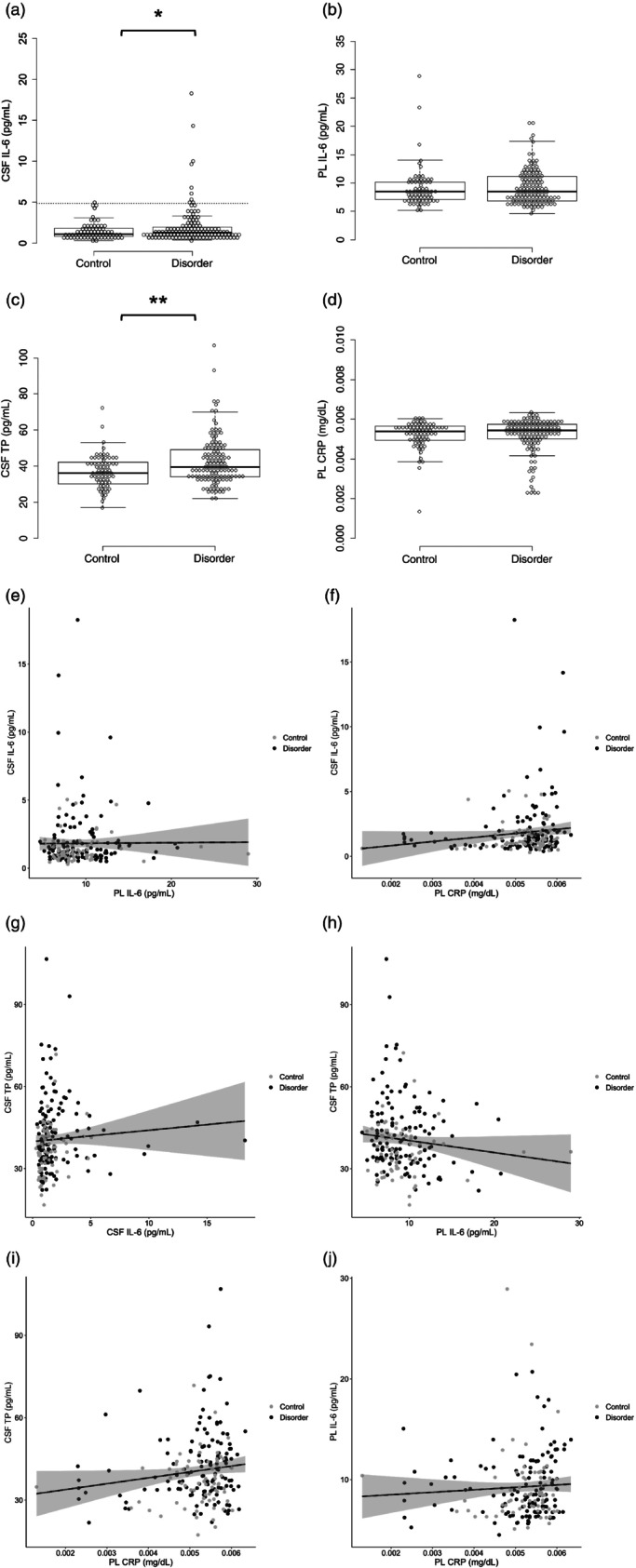
IL‐6, total protein, and CRP levels in participants. (a–d) Dot plots of molecular levels in CSF and plasma. (a) The dotted line represents the 97.5% value of the CSF IL‐6 levels in the control group (4.85 pg./mL). (e–j) Scatter plots of molecular levels in CSF and plasma. Black and gray dots represent the psychiatric patients and controls, respectively. Straight line in the figure indicates the regression line, and the shaded area delineates the 95% confidence interval. *P < 0.1, **P < 0.05.

CSF and plasma IL‐6, plasma CRP, and CSF TP levels in the patients and controls are shown in Table 1 and Fig. 1a–d. There was a nearly‐significant difference (*P* = 0.055) in CSF IL‐6 levels between the patients and controls. The mean CSF TP levels were significantly higher in the patients than in the controls (*P* = 0.002). Plasma IL‐6 and CRP levels were not normally distributed, and their levels did not differ significantly between the two groups. There was no significant difference in CSF or plasma IL‐6, or in plasma CRP levels among the diagnostic groups (*P* > 0.1, data not shown).

**Fig. 2:**
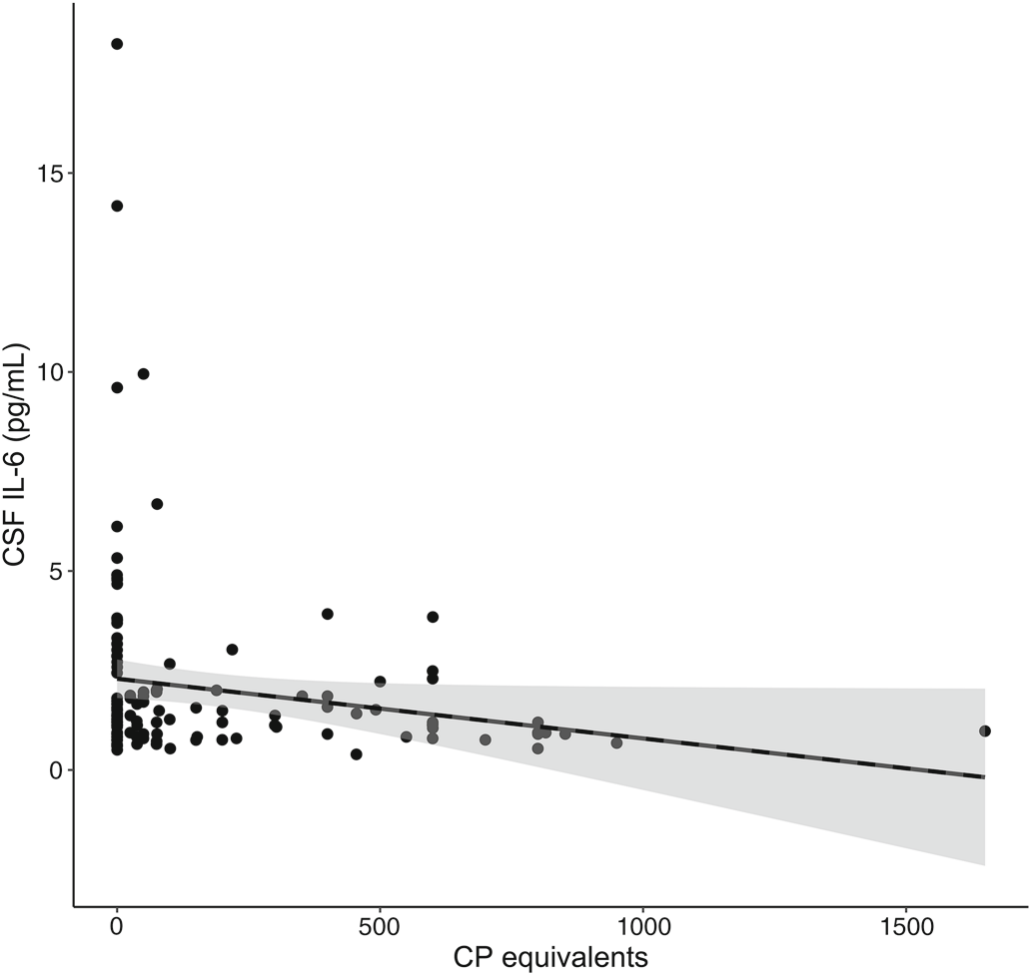
Scatter plot of CSF IL‐6 levels and chlorpromazine (CP) equivalents of antipsychotics. Straight line in the figure indicates the regression line, and the shaded area delineates the 95% confidence interval.

The CSF and plasma IL‐6 levels were not significantly correlated (rs = 0.045, *P* = 0.53; Fig. 1e). The CSF IL‐6 levels had a significant positive correlation with plasma CRP levels (rs = 0.157, *P* = 0.028; Fig. 1f). CSF TP levels had significant positive correlations with CSF IL‐6 levels (rs = 0.24, *P* < 0.001; Fig. 1g) and plasma CRP levels (rs = 0.14, *P* = 0.049; Fig. 1i). Plasma IL‐6 levels were not significantly correlated with CSF TP or plasma CRP levels (Fig. 1h,j). CSF IL‐6 and antipsychotic chlorpromazine equivalents showed a significant negative correlation (rs = −0.21, *P* = 0.017; Fig. 2). Equivalents of antidepressant and benzodiazepines were not significantly correlated with CSF or plasma IL‐6 levels. ANCOVA (controlling for age and sex) revealed no significant differences in demographic data or molecule levels between patients not taking psychotropic medications and controls.

### Correlation between CSF IL‐6 levels and psychological scales

**Fig. 3:**
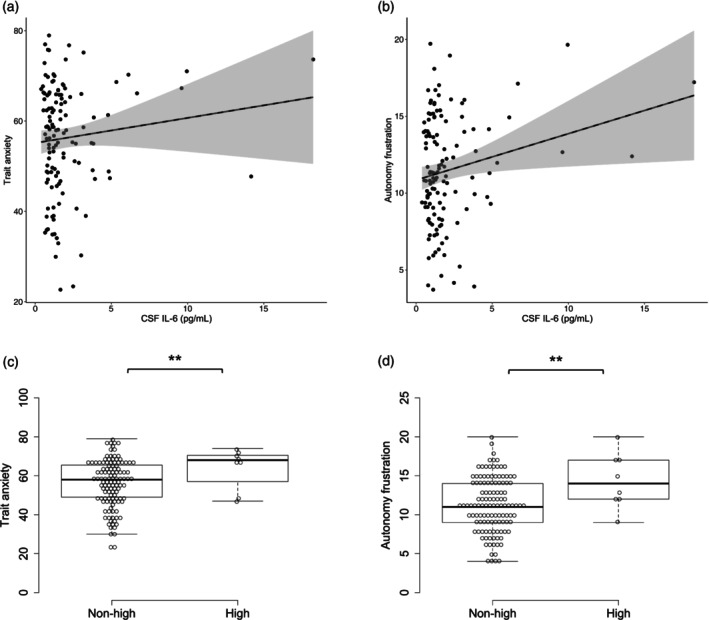
CSF IL‐6 levels and mental state examinations of patients. Correlation of CSF IL‐6 levels with STAI (State–Trait Anxiety Inventory) trait anxiety (a) and BPNFSF (Basic Psychological Need Satisfaction and Frustration Scale) autonomy frustration (b) scores. Straight line in the figure indicates the regression line, and the shaded area delineates the 95% confidence interval. (c) and (d) Dot plots of psychological scales for high and non‐high CSF IL‐6 with disorders. **P < 0.05.

Although age had no significant correlation with CSF IL‐6 levels, we found that CSF IL‐6 levels were significantly higher in males than in females (*P* < 0.001), and that BMI had a significant positive correlation with CSF IL‐6 levels (rs = 0.25, *P* < 0.001). The partial correlation analysis, controlling for age, sex, BMI and JART, showed that CSF IL‐6 levels had a significant positive correlation with STAI‐T (*r* = 0.25, *P* = 0.046) and BPNSFS‐autonomy frustration (AF) (*r* = 0.29, *P* = 0.018) (Fig. 3a,b).

CSF IL‐6 and PANSS, MADRS, and YMRS showed no significant correlation. Additionally, there was no significant correlation between CSF‐IL‐6 and psychiatric symptoms when analyzed separately for each psychiatric disorder. Among the psychological symptoms and disease severity scales, significant correlations were found between the STAI‐T and BPNSFS‐AF (rs = 0.45, *P* < 0.001). STAI‐T was significantly correlated with MADRS (rs = 0.52, *P* < 0.001) and PANSS total score (rs = 0.45, *P* < 0.001). BPNSFS‐AF was significantly correlated with MADRS (rs = 0.34, *P* < 0.001), but not with PANSS or YMRS scores.

### Participants with abnormally high CSF IL‐6 levels

Based on the above observation, we then compared patients with abnormally high CSF IL‐6 levels and those with non‐high IL‐6 levels. The cut‐off for separating the abnormally high and non‐high groups was set at the 97.5 percentile value of the controls (4.85 pg./mL). The clinical characteristics of the patients with high CSF IL‐6 levels in CSF are shown in Table 2 (mean age 35.7 ± 9.2, male 71.4%, BMI 23.2 ± 4.4). Two patients were drug‐free for at least 2 weeks, and the other participants were medicated with antidepressants (*n* = 4), mood stabilizers (*n* = 2), antipsychotics (*n* = 2), and benzodiazepines (*n* = 4). Five out of the eight patients had a history of treatment resistance or discontinuation of antidepressants due to adverse effects according to medical records.

**Table 2:** Clinical characteristics of participants with high IL‐6 level in CSF

Case	Age	Sex	Clinical diagnoses	BMI (kg/m^2^)	CSF IL‐6 (pg/mL)	Prescriptions
1	31	Male	Depression	21.4	18.24	None
2	44	Male	Schizophrenia	21.7	14.17	None
3	45	Male	Depression	26.4	9.95	Valproic acid, escitalopram, risperidone, cloxazolam, ramelteon
4	43	Female	Depression	21.9	9.61	Paroxetine, ethyl loflazepate, brotizolam
5	21	Female	Bipolar disorder	18.6	6.68	Valproic acid, sertraline, quetiapine, lorazepam, flunitrazepam, zolpidem
6	38	Male	Depression	21.0	6.12	Venlafaxine, tandospirone, etizolam, zolpidem, ramelteon
7	28	Male	Depression	31.7	5.33	Bromazepam, eszopiclone, zolpidem
8	63	Male	Control	27.7	5.05	–
9	54	Male	Bipolar disorder	23.1	4.90	Clomipramine, milnacipran, suvorexant, triazolam

Abbreviations: BMI, body mass index; CSF, cerebrospinal fluid.

**Fig. 4:**
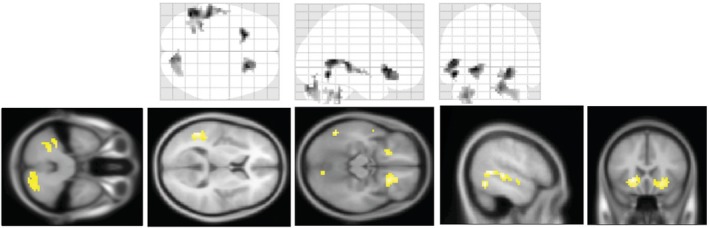
Regions with significant differences in rCBF between patients with high CSF IL‐6 levels and the remaining patients.

Patients with high CSF IL‐6 levels had significantly higher STAI‐T scores (*P* = 0.035) and BPNSFS‐AF scores (*P* = 0.026) than the remaining patients (Fig. 3c,d). In ASL analysis, patients with high CSF IL‐6 levels had significantly higher rCBF in the left superior temporal gyrus, bilateral nucleus accumbens (NAc), and cerebellum than the remaining patients, controlling for age and sex (Fig. 4 and Table 3). CSF IL‐6 levels and rCBF did not show a significant correlation.

**Table 3:** Brain regions with significant differences in rCBF between patients with high and normal CSF IL‐6 levels

Brain Region	Cluster size	Z score	x	y	z
Left superior temporal region	194	4.42	−51	−31	1
Left nucleus accumbens	81	4.32	−21	20	−5
Right nucleus accumbens	112	4.19	12	20	−8
Right cerebellum	166	3.97	12	−85	−29
Left cerebellum	117	3.77	−33	−43	−38

## Discussion

We found that CSF IL‐6 levels were significantly correlated with trait anxiety and autonomy frustration in patients with psychiatric disorders. Elevations in CSF IL‐6 are widely observed in neuroinflammation and are reported to correlate with the prognosis of neurological diseases such as traumatic brain injuries,^30^, ^31^ multiple sclerosis,^32^ and neuro‐Behçet's syndrome.^33^, ^34^ Elevated CSF IL‐6 levels have also been reported in psychiatric disorders, such as mood disorders,^1^, ^2^ schizophrenia,^1^, ^3^ and post‐traumatic stress disorder,^4^ whereas conflicting findings have also been reported.^35^, ^36^ We hypothesized that CSF IL‐6 is associated with psychiatric symptoms across psychiatric disorders, and examined whether CSF IL‐6 is associated with anxiety and frustration, which are common symptoms in psychiatric disorders and components of NVS. In the present results, CSF IL‐6 levels were associated with trait anxiety and autonomy frustration. Trait anxiety in the STAI‐T is suggested to be not only a personal general tendency to experience anxiety in situations but also a nonspecific measure of negative affect.^37^ Trait anxiety is also suggested to be associated with the risk of depression^38^, ^39^ and psychotic relapse in schizophrenia.^40^ Autonomous frustration in the BPNSFS refers to the experience of feeling controlled, either externally enforced or self‐imposed,^19^ and it is also associated with non‐reward frustration and amotivation.^41^ Our results suggest that neuroinflammation with elevated CSF IL‐6 levels may be a common factor of psychiatric disorders that is associated with anxiety and frustration, the commonly observed symptoms in psychiatric disorders. Furthermore, trait anxiety was significantly correlated with MADRS and PANSS scores, and the autonomous frustration was also significantly correlated with the MADRS, suggesting that trait anxiety may be associated with severity of psychotic and depressive symptoms and frustration may be associated with severity of depressive symptoms.

In addition, eight patients above the CSF IL‐6 cut‐off levels also had higher scores of trait anxiety and autonomy frustration than the rest of the patients, and their rCBFs were increased in the NAc, left temporal gyrus, and cerebellum. The NAc is a major element implicated in reward and motivation,^42^ and correlations with trait anxiety have been suggested.^43^, ^44^ Autonomous frustration is associated with non‐reward frustration, and may be related to increased activity in the NAc, which is known to be activated by abstinence‐induced cigarette craving in chronic smokers,^45^ and by drug craving in cocaine addiction,^46^ which is often induced by frustration. Alterations in rCBF have been reported in neuroinflammatory disorders such as neuropsychiatric systemic lupus erythematosus^47^ and anti‐NMDAR encephalitis.^48^ Taken together, high CSF IL‐6 levels associated with anxiety and frustration, may be related to altered rCBF of specific brain regions. However, CSF IL‐6 levels and rCBF did not show a significant correlation. While the overall correlation was not significant, it is possible that abnormally high CSF IL‐6 levels could affect brain and increase CBF. Further, although we focused on anxiety and frustration, a possibility remains that CSF IL‐6 levels may be related with other psychopathological components, which requires further investigations.

In the present study, CSF IL‐6 levels showed a significant negative correlation with antipsychotic equivalent doses, suggesting that antipsychotics may suppress CSF IL‐6 levels. Additionally, two of the eight patients with high CSF IL‐6 levels were drug free. The anti‐inflammatory effect of antipsychotics has been suggested,^49^ which is consistent with our result. Besides, anti‐inflammatory treatments have been suggested to be effective to treat psychiatric symptoms,^50^, ^51^ including IL‐6 antibody therapies.^52^, ^53^, ^54^ In contrast, opposite results have also been reported. Knight *et al*. reported that blockade of IL‐6 receptors with tocilizumab significantly worsened depressive symptoms in medically ill patients.^55^ Girgis *et al*. reported that tocilizumab had no effect on residual symptoms in patients with schizophrenia.^56^ The inconsistencies among previous reports on the effects of anti‐inflammatory therapy may be due to differences in the proportion of patients with elevated CSF IL‐6 levels included in the studies. Anti‐inflammatory treatments may be more effective in selected patients based on their CSF IL‐6 levels.

In psychiatric disorders and inflammatory conditions, increased peripheral blood cytokine levels are thought to alter BBB permeability and lead to neuroinflammation.^51^, ^57^ Qin *et al*. reported that systemic administration of LPS in mice caused chronic neuroinflammation.^58^ Engler *et al*. reported a selective increase in CSF IL‐6 levels after LPS injection in healthy participants.^6^ Thus, we analyzed the association between CSF IL‐6 and peripheral paired marker levels. However, the CSF and plasma IL‐6 levels were not significantly correlated. Additionally, peripheral IL‐6 levels were not correlated with CSF TP levels, an indicator of BBB integrity. A recent systematic review and meta‐analysis suggested that peripheral inflammatory markers poorly reflect paired CSF markers.^59^ One possible explanation for this discrepancy is that there may be a latency period before peripheral inflammation alters BBB integrity, leading to neuroinflammation. CSF IL‐6 levels may continue to increase even after the peripheral inflammation subsides.^6^, ^58^ Accordingly, long‐term effects of peripheral inflammation on neurons have been suggested.^60^ Interestingly, plasma CRP was positively correlated with CSF IL‐6 and CSF TP in our study, whereas plasma CRP and plasma IL‐6 were not. Consistent with our findings, a recent study has shown a correlation between plasma CRP and CSF inflammatory markers, including IL‐6, suggesting the potential of plasma CRP as a peripheral biomarker of neuroinflammation.^61^ Although plasma CRP levels did not exceed the clinically significant threshold in our study, this may reflect an unknown relationship between peripheral inflammation and neuroinflammation.

There were several limitations in the present study. First, the observed mean CSF IL‐6 levels between the patients and controls showed a difference at the trend level but did not reach statistical significance. This may be due to the inadequate statistical power by the small sample size of the study. In addition, the majority of the patients were medicated, and as described above, antipsychotic medication, in particular, may have reduced the difference in CSF IL‐6 levels between the patients and controls. Second, the comparison between patients with abnormally high CSF IL‐6 levels and those with non‐high IL‐6 levels was a post‐hoc analysis involving a small number of patients with abnormally high levels. Controls were not given psychological assessments, so it is unclear if the CSF IL‐6 and anxiety/frustration association is disease‐specific. Future research with larger sample size should include psychological assessments in the control group to further explore this relationship. Another limitation was the cross‐sectional design, and longitudinal studies will clarify whether CSF IL‐6 levels are state‐dependent or persistent. In addition, the multiplicity of analyses was not adequately corrected for, indicating the exploratory nature of the present study. However, our results warrant future psychiatric research beyond the existing diagnostic frameworks and may contribute a small step in elucidating the etiology of psychiatric disorders.

## Conclusions

We found that high CSF IL‐6 levels were associated with anxiety and frustration across psychiatric disorders, which may be related to altered CBF of specific brain regions. Elevated CSF IL‐6 level is a potential marker that may reflect the neuroinflammatory subpopulation and basic negative valence symptoms across psychiatric disorders.

## Disclosure statement

H.K received grants or contracts from AMED (20356139), Kakenhi B (22544910), and Kakenhi C (21359395) in the past 36 months; payment/honoraria from Yakult Honsha Co., Ltd., Nobelpharma Co. Ltd., Meiji holdings Co. Ltd., Otsuka Pharmaceutical Co., and Meiji‐Seika Pharma Co. in the past 36 months; payment for expert testimony from Ajinomoto Co. Ltd., in the past 36 months; and has US patent 1,174,317 in the past 36 months. K.N received grants from SHIONOGI & Co., Ltd., Sumitomo Pharma Co., Ltd., Otsuka Pharmaceutical Co., Meiji‐Seika Pharma Co., Ltd., Janssen Pharmaceutical K.K., Mitsubishi Tanabe Pharma Corp., Nippon Boehringer Ingelheim Co., Ltd., and MOCHIDA PHARMACEUTICAL CO., LTD. in the past 36 months; payment/honoraria from Sumitomo Pharma Co., Ltd., Otsuka Pharmaceutical Co., Meiji‐Seika Pharma Co., Ltd., Janssen Pharmaceutical K.K., Mitsubishi Tanabe Pharma Corp., Takeda Pharmaceutical Co., Ltd., Lundbeck Japan, Viatris Inc., Eisai Co., Ltd., Nippon Boehringer Ingelheim Co., Ltd., and MOCHIDA PHARMACEUTICAL CO., LTD. in the past 36 months; and support for attending meetings and/or travel from Sumitomo Pharma Co., Ltd., Otsuka Pharmaceutical Co., Meiji‐Seika Pharma Co., Ltd., Janssen Pharmaceutical K.K., Mitsubishi Tanabe Pharma Corp., Takeda Pharmaceutical Co., Ltd., Nippon Boehringer Ingelheim Co., Ltd., and MOCHIDA PHARMACEUTICAL CO., LTD. in the past 36 months.

## Author contributions

Conceptualization: K. H., H. K., and K. N.; methodology: M. O. and M. T.; formal analysis: T. E., K. O., S. O., and K. H.; investigation: T. E.; resources: T. E., K. H., N. S., and M. T.; writing—original draft preparation: T. E.; writing—review and editing: K. H.; supervision: T. N., S. O., M. H., and K. N.; funding acquisition: K. H., T. N., and K. N. All authors read and agreed to the published version of the manuscript.

## Funding information

This study was supported by an Intramural Research Grant (3‐1) for Neurological and Psychiatric Disorders of the NCNP from K.H., T.N., and K.N.

## Supporting information


**Table S1.** Disease severity and daily doses of psychotropics in patients with psychiatric disorders.
