# Supplementary material for: Possible association of elevated CSF IL‐6 levels with anxiety and frustration in psychiatric disorders
Source: Psychiatry Clin Neurosci. 2024 Sep 24;78(12):792–9. doi: 10.1111/pcn.13743 (PMC11612533; doi:10.1111/pcn.13743)
Supplement: Supplementary file 1 — Table S1. Disease severity and daily doses of psychotropics in patients with psychiatric disorders. [file PCN-78-792-s001.docx]

**Supplementary Table 1.** Disease severity and daily doses of psychotropics in patients with psychiatric disorders

|  | All  129 | Depression  49 | Bipolar disorder  31 | Schizophrenia  30 | Other  19 |
| --- | --- | --- | --- | --- | --- |
| Total PANSS scores | 49  (41–62) | 44  (40–53) | 48  (40.5–51.5) | 65  (58–73) | 54  (37.5–63) |
| MADRS | 14  (6–22) | 16  (7–23) | 16  (11–19.5) | 10  (6–16) | 10  (2–17) |
| YMRS | 0  (0–1) | 0  (0–0) | 2  (0–4.5) | 0  (0–1) | 0  (0–0) |
| Antipsychotics | 25  (0–209) | 0  (0–50) | 50  (0–125) | 400  (100–600) | 0  (0–12.5) |
| Antidepressants | 0  (0–119) | 150  (38–200) | 0  (0–75) | 0  (0–0) | 0  (0–0) |
| Benzodiazepines | 5  (0–11.9) | 8  (2–16.3) | 10  (2.3–13.8) | 5  (0–15) | 1.3  (0–4.6) |

Median severity and medication dosage. Interquartile ranges are shown in parentheses. Daily doses of antipsychotics, antidepressants, and benzodiazepines were converted into equivalent doses of chlorpromazine, imipramine, and diazepam, respectively.
